# Identification of Splicing Quantitative Trait Loci (sQTL) in *Drosophila melanogaster* with Developmental Lead (Pb^2+^) Exposure

**DOI:** 10.3389/fgene.2017.00145

**Published:** 2017-10-24

**Authors:** Wen Qu, Katherine Gurdziel, Roger Pique-Regi, Douglas M. Ruden

**Affiliations:** ^1^Laboratory of Epigenomics, Department of Pharmacology, C.S. Mott Center for Human Growth and Development, Wayne State University, Detroit, MI, United States; ^2^Department of Obstetrics and Gynecology, Wayne State University, Detroit, MI, United States; ^3^Center for Molecular Medicine and Genetics, Wayne State University, Detroit, MI, United States; ^4^Institute of Environmental Health Sciences, Wayne State University, Detroit, MI, United States

**Keywords:** developmental lead exposure, *Drosophila melanogaster*, splicing quantitative trait loci (sQTL or splice QTL), *Drosophila* synthetic population resource (DSPR), RNA-seq, toxicogenomics

## Abstract

Lead (Pb) poisoning has been a major public health issue globally and the recent Flint water crisis has drawn nation-wide attention to its effects. To better understand how lead plays a role as a neurotoxin, we utilized the *Drosophila melanogaster* model to study the genetic effects of lead exposure during development and identified lead-responsive genes. In our previous studies, we have successfully identified hundreds of lead-responsive expression QTLs (eQTLs) by using RNA-seq analysis on heads collected from the *Drosophila* Synthetic Population Resource. *Cis*-eQTLs, also known as allele-specific expression (ASE) polymorphisms, are generally single-nucleotide polymorphisms in the promoter regions of genes that affect expression of the gene, such as by inhibiting the binding of transcription factors. *Trans*-eQTLs are genes that regulate mRNA levels for many genes, and are generally thought to be SNPs in *trans*-acting transcription or translation factors. In this study, we focused our attention on alternative splicing events that are affected by lead exposure. Splicing QTLs (sQTLs), which can be caused by SNPs that alter splicing or alternative splicing (AS), such as by changing the sequence-specific binding affinity of splicing factors to the pre-mRNA. We applied two methods in search for sQTLs by using RNA-seq data from control and lead-exposed w^1118^ *Drosophila* heads. First, we used the fraction of reads in a gene that falls in each exon as the phenotype. Second, we directly compared the transcript counts among the various splicing isoforms as the phenotype. Among the 1,236 potential Pb-responsive sQTLs (*p* < 0.0001, FDR < 0.39), mostly *cis*-sQTLs, one of the most distinct genes is *Dscam1* (Down Syndrome Cell Adhesion Molecule), which has over 30,000 potential alternative splicing isoforms. We have also identified a candidate Pb-responsive *trans*-sQTL hotspot that appears to regulate 129 genes that are enriched in the “cation channel” gene ontology category, suggesting a model in which alternative splicing of these channels might lead to an increase in the elimination of Pb^2+^ from the neurons encoding these channels. To our knowledge, this is the first paper that uses sQTL analyses to understand the neurotoxicology of an environmental toxin in any organism, and the first reported discovery of a candidate *trans*-sQTL hotspot.

## Introduction

### Lead burdens

Although, the phase-out of leaded paint and gasoline has substantially reduced mean blood lead levels in the United States (White et al., 2007), lead contamination in the city of Detroit and the neighboring city of Flint have been of extreme concern in the past two years due to the Flint water crisis (Hanna-Attisha et al., 2015). In a recent study, we found multigenerational epigenetic effects on DNA methylation in Detroit children (Senut et al., 2012; Sen et al., 2015a,b). Additionally, lead exposure from environmental contamination remains a major global public health issue (Tong et al., 2000; Rauh and Margolis, 2016).

Our lab has been studying the genetics of lead neurotoxicology using the *Drosophila melanogaster* model (Hirsch et al., 2012). In our genetic and physiology studies, 250 μM lead acetate in the standard fly food causes adult soft-tissue lead levels to be 50–100 μg/dL (Hirsch et al., 2009). This is in the high range of human lead exposure, and the currently CDC level of concern for lead exposure is 5 μg/dL (Bellinger, 2013). We found that lower levels of lead exposure, as low as 50 μM, can significantly alter the *Drosophila* larval neuromuscular junction (Morley et al., 2003; He et al., 2009a,b). In the current studies, we use 250 μM developmental exposure to lead in the food because it consistently affects synaptic (He et al., 2009a), behavioral (Hirsch et al., 2009), and gene expression changes (Ruden et al., 2009). In our previous *Drosophila* analyses, we found that lead exposure modified the uniformity of the synaptic matching between axons and muscle fibers during larval development (Morley et al., 2003) and it also changes a variety of behavioral phenotypes such as courtship (Hirsch et al., 1995) and circadian locomotor activity (Hirsch et al., 2003). Additionally, our lab has used microarrays and RNA-seq technology along with eQTL analysis to map lead-sensitive genes in *Drosophila* (Ruden et al., 2009). Therefore, we are confident that *Drosophila* is a useful model to understand some of the mechanisms for how developmental lead exposure to lead affects gene expression and splicing in neurons, some of which are likely to be conserved in humans.

### Alternative splicing

After the discovery of splicing in the Adenovirus *hexon* gene in 1977 (Sambrook, 1977), Walter Gilbert proposed in 1978 that different combinations of exons and introns, namely “alternative splicing” (AS), could produce different mRNA isoforms of a gene (Gilbert, 1978; Modrek and Lee, 2002). The disparity between the expected 150,000 or more human genes and the surprising actual report of under 32,000 later suggested an underestimated role for alternative splicing in the production of an increased variety of mRNAs and proteins (Pennisi, 2000; Venter et al., 2001). It has been estimated that AS is a crucial form of gene regulation affecting about 60–90% of human genes (Modrek and Lee, 2002) and over 40% of *Drosophila* genes (Stolc et al., 2004). Mutations that affect mRNA splicing and AS were also considered to be highly linked with disease occurrences (Singh and Cooper, 2012). In addition, it has been estimated that 15% of human disease mutations lie within splicing sites and 22% of disease-related SNPs may affect splicing (Krawczak et al., 2007; Lim et al., 2011).

The *Drosophila Dscam1* gene exemplifies one of the most extreme examples of alternative splicing. *Dscam1* (Down Syndrome Cell Adhesion Molecule 1) is a cell surface protein which gives rise to over 30,000 potential alternatively spliced isoforms in the *Drosophila* nervous system (Schmucker and Flanagan, 2004). The human homolog *DSCAM* was first discovered as a candidate disease gene for the central and peripheral nervous system defects associated with Down syndrome (Yamakawa et al., 1998). The *Drosophila Dscam1* was later found to have extreme structural diversity and is essential for neural circuit assembly (Schmucker et al., 2000; Hattori et al., 2007). Its diversity allowed each neuron to have a unique pattern on its cell membrane, which made self-recognition possible (Zipursky and Grueber, 2013; Armitage et al., 2015). It has also been shown that *Dscam1* regulated interactions between neurons through isoform-specific homophilic binding or repulsion (Wojtowicz et al., 2004; Tadros et al., 2016). Additionally, its role in the insect cellular immune system has been suggested since 2005 (Watson et al., 2005). Even after years of study, many questions remain unanswered, such as how *Dscam1* mRNA isoforms are selectively expressed and how homophilic interactions are translated into binding or repulsing responses during neurogenesis (Schmucker and Flanagan, 2004).

### Quantitative trait locus

A quantitative trait locus (QTL) is a sequence of DNA (the locus) that is associated with variation in a phenotype (the quantitative trait) (Miles and Wayne, 2008). In the case of expression QTLs (eQTLs) and splicing QTLs (sQTLs) (Yoo et al., 2016; Mei et al., 2017; Takata et al., 2017; Yang et al., 2017), the quantitative traits are relative gene expression levels and relative splicing isoform abundance. All QTLs, no matter the type, are typically distributed in a normal distribution in the population. Significant eQTLs and sQTLs could be categorized into two groups: *cis*- and *trans*-eQTLs and sQTLs. Here, in this paper, we focus on *cis*- and *trans*- sQTLs. By definition, *cis*-sQTLs are referred as genetic variants that affect the splicing event of a locus only on the same haplotype, while *trans*-sQTLs affect multiple haplotypes (Benzer, 1955; Hasin-Brumshtein et al., 2014). Therefore, *cis*-sQTLs tend to be “local,” adjacent to the transcript location, while *trans*-sQTLs tend to be “distal,” away from the regulator (Benzer, 1955; Hasin-Brumshtein et al., 2014). For the purposes of this paper, an sQTL is defined as genetic variants that are associated with changes in the splicing ratios of transcripts (Monlong et al., 2014).

In our previous studies, we described the identification of *cis*- and *trans*-eQTLs in *Drosophila*. We identified lead-responsive *cis*-eQTLs and *trans*-eQTLs by using Affymetrix *Drosophila* gene expression microarrays in 2009 (Ruden et al., 2009). At that time, we also found eQTLs linked with developmental behavioral effects of lead exposure (Hirsch et al., 2009). In a more recent study, we used RNA-seq technology to quantify expression profiles and ran a similar analysis to map lead–sensitive eQTLs in *Drosophila*. We used *Drosophila* samples that may include more genetic variations to further explore the mechanisms of the Pb-responsive *cis*- and *trans*-eQTLs (manuscript submitted to Frontiers in Genetics). Here, in this paper, we use the same set of RNA-seq data to search for Pb-responsive sQTLs. We found hundreds of candidate *cis*-sQTLs, and one major *trans*-sQTL hotspot, among which *Dscam1* was one of the most significant *cis*-sQTLs both at the exon level and at the transcript level.

## Materials and methods

### Genotyping

The 79 *D. melanogaster* sample lines were from one set of *Drosophila* Synthetic Population resource (DSPR)—Subpopulation A2 (http://FlyRILs.org) and the genomic data were downloaded from http://wfitch.bio.uci.edu/R/ (King et al., 2012). This eight-way synthetic population was first initiated with eight inbred founder lines (A1–A8) with a wide genetic variation. The first generation was by intercrossing A1 with A2 and A2 with A3 until A7 with A8. 10 offspring per genotype per sex were mixed together to establish the next generation. After 50 generations of intercrossing and 25 generations of inbreeding, ~1,600 recombinant inbred lines (RILs) were generated. The Restriction-Associated DNA (RAD) and hidden Markov model (HMM) were used to estimate the genetic contribution of parental lines in each RIL and the genetic data contained the complete set of underlying founder haplotype structure for all RILs (King et al., 2012). All the genotype information was provided by the DSPR group (King et al., 2012).

### Sample collection

Flies were cultured at 25°C in 35 ml vial containing standard *Drosophila* 10 ml medium. Medium was blended with 250 μM PbAc or 250 μM NaAc in order to mimic lead toxicity (Hirsch et al., 2009). Total RNA was extracted from 50 adult male heads (5–10 days old) of DSPR homozygous recombinant inbred lines by using TruSeq Cluster RNA sample prep kit provided by Illumina and 1 μg of RNA was used after RNA isolation. The D1K ScreenTape on the Agilent TapeStation instrument along with the quantitative PCR on the QuantStudio 12K Flex was used to guarantee the quality of library. Libraries were standardly prepared and sequenced on the Hiseq2000™ instrument from Illumina (50-bp paired end reads). General read quality was examined by FastQC (Andrews, 2010). The average coverage is 23 million read pairs and the RNA-seq data are available on the NCBI GEO accession: GSE83141.

### Expression quantification

RNA-seq reads were mapped to the UCSC/dm3 *D. melanogaster* references genome (track: Flybase Genes) using TopHat (Karolchik et al., 2004; Kim et al., 2013). Upload/Convert ID tool from Flybase.org was used to convert the annotation symbols into official symbols (Tweedie et al., 2009) and Htseq was used to quantify exon and transcript expression (Anders et al., 2014). The RIL information was used as a covariate (Y ~ treatment + RIL), when performing the differential expression analysis. For the GO enrichment analysis, GOseq was used to search among the differentially expressed genes after lead exposure and genetic programming (GP) categories of “Molecular Function” and “Biological Process” were selected (Kent et al., 2002; Young et al., 2010).

### ANOVA test

All analyses were performed in R-3.2.3. After calculating division of exon reads to its corresponding transcript reads, quantile normalization, and confounding factors were removed by PCA (n.pc = 3), we used the following test to identify sQTLs for each transcript:

$$\begin{array}{l} \text{H}0:\;Y_{n}=\mu +\beta^{E}E_{n}+\epsilon_{n}\\ \text{HA}:\;Y_{n}=\mu +\underset{i}{\sum }\left(\beta_{i}^{G}G_{ij}+\beta_{i}^{G\times E}G_{ij}E_{n}\right)+\beta^{E}E_{n}+\epsilon_{n} \end{array}$$

where *Y*_*n*_ is the vector of splicing measures, *G*_*ij*_ is the i-th parental genotype probability at locus j, *E*_*n*_ is a vector representing two environmental conditions (control or lead-treated). The parameters μ, β^*E*^, β^*G*^, β^*G*×*E*^ represent respectively the grand mean, the genotype, the environment and the interaction effects.

For transcript expression levels, transcript reads were quantile normalized, and confounding factors were removed by PCA (n.pc = 4). Expression data for genes that have more than one isoform were subgrouped (max. 3,975). Then for each gene we test for interaction between G × E interaction in splicing isoform usage using the following model:

$$\begin{array}{l} \text{H}0:\;Y_{nk}=\mu_{k}+\underset{ik}{\sum }\left(\beta_{ik}^{G}G_{ij}\right)+\underset{k}{\sum }\left(\beta_{k}^{E}T_{k}E_{n}\right)+\epsilon_{nk}\\ \text{HA}:\;Y_{nk}=\mu_{k}+\underset{ik}{\sum }\left(\beta_{ik}^{G}G_{ij}T_{k}+\beta_{ik}^{G\times E}T_{k}G_{ij}E_{n}\right)\\ \;+\underset{k}{\sum }\left(\beta_{k}^{E}T_{k}E_{n}\right)+\epsilon_{nk} \end{array}$$

where *Y*_*nk*_ is a matrix representing all normalized read counts across individuals *n* and isoforms *k* for each gene, *G*_*ij*_ is the i-th parental genotype probability at locus j, *E*_*n*_ is a vector representing two environmental conditions (control or lead-treated), *T*_*k*_ is an indicator variable for each isoform *k*. The parameters μ_*k*_, $\beta_{k}^{E}$, $\beta_{ik}^{G}$, $\beta_{ik}^{G\times E}$, represent respectively the grand mean, the genotype, the environment and the interaction effects across different isoforms.

After obtaining all the *p*-values indicating the likelihood of association between each genomic location and each transcript, *q*-value function in R (library: *q*-value) was used to transform the *p*-values into FDRs and we defined *p* ≤ 0.0001, corresponding FDRs ≤ 0.39, as significant signals.

## Results

### Differential expression modification after lead exposure

**Figure 1:**
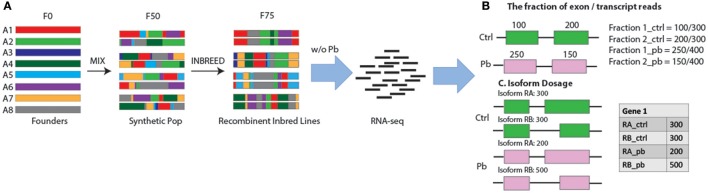
Workflow in search for sQTLs. **(A)** The design of the recombinant inbred lines. Strains were initiated with eight founder strains A1–A8 with a diverse geographic origin. In the first generation, lines were intercrossed with each other and 10 F1 flies per genotype per sex were mixed together to establish the next generation. This mix went on until the 50th generation when flies were separated and Inbreeding continued for another 25 generations, leading to a total of ~1,600 completed recombinant inbred lines. After samples were treated w/o Pb, RNA-seq analysis was performed and two methods were used to target sQTLs: **(B)** the fraction of exon reads to transcript reads, and **(C)** Isoform dosage.

In order to search for sQTLs, we collected RNA-seq data from 79 fly lines from The *Drosophila* Synthetic Population Resource (DSPR) (King et al., 2012). The lines were started with eight founder strains (A1–A8) that come from diverse demographic origins and include several million genetic variations in the *D. melanogaster* species. The eight founder lines were intercrossed for 50 generations and were separated to inbred for additional 25 generations (Figure 1A) (King et al., 2012). We randomly selected 79 lines out of the ~1,600 recombinant inbred lines and treated them with standard fly food plus 250 μM NaAc or 250 μM PbAc. Fifty heads of male flies in each sample were collected manually by forceps and dropped into RNALater™, and RNA-seq was performed per the standard protocol (see section Materials and Methods). Thus, we had 158 RNA-seq samples (79 ^*^2 ± Pb).

**Figure 2:**
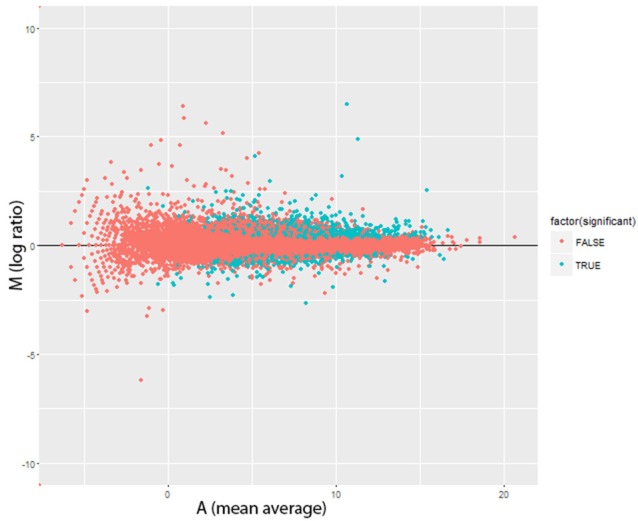
Altered Gene Expression Levels after Lead Treatment in *Drosophila* male head. MA plots for change in transcript expression (*n* = 1,682) comparing Pb-treated (*n* = 79) with control-treated samples (*n* = 79). Red dots represent transcript expression profiles were not significantly changed and cyan dots were significantly regulated transcripts (FDR < 0.0001, 0.450 ± 0.272 mean log2 fold changes ± s.d). M = log2(P/C), A = (log2(C)+log2(P))/2 (P, Pb-treated FPKM values; C, control FPKM values).

**Figure 3:**
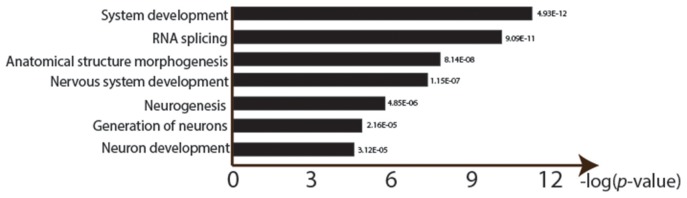
Gene ontology enrichment analysis of lead treatment in *Drosophila* male heads. GOseq was used to detect over represented GO categories in RNA-seq data (FDR < 0.0001). Y-axis shows the logarithm of each significant GO ID's *p*-value (after Bonferroni correction for multiple testing).

There were significant changes in the gene expression profiles after lead exposure: 1,682 changes among the 20,507 transcripts (14,058 genes), including 187 exhibiting over 50% change in expression levels (FDR < 0.0001, 0.450 ± 0.272 mean absolute log_2_ change ± s.d.; Figure 2; Supplemental Table 1). Among the responders, 1,338 transcripts were upregulated after lead treatment and 344 transcripts were downregulated. Among all the significantly regulated transcripts, nervous system development and neurogenesis were among the topmost enriched gene ontology (GO) categories (Figure 3). These results correspond with our expectations, since during sample preparation, we only collected *Drosophila* heads and the nervous system development has been regarded as the main target for Pb toxicity (Baranowska-Bosiacka et al., 2012).

### Identification of *cis*-splicing QTLs

After detecting differentially expressed transcripts affected by Pb treatment, we continued to search for splicing QTLs—the genomic regions where genetic variants affect splicing events. In most sQTL studies (Lappalainen et al., 2013; Battle et al., 2014; Kurmangaliyev et al., 2015; Ongen and Dermitzakis, 2015), SNPs were used to reflect the genomic variations. However, in our study, each fly line was a mosaic of the eight parental lines (A1–A8; Figure 1A) and the genetic contribution by the parental genotypes represent the genomic variations. With this type of genotype information, the cis-sQTL is defined as a genomic locus where alternative splicing is associated with a differential parental contribution.

Reads were mapped by Tophat2 (Trapnell et al., 2012; Kim et al., 2013) and quantified to exons and transcripts by Htseq (Anders et al., 2014). Our major goal in this paper is to identify splicing QTLs. However, our RNA-seq data is 50 bp paired-end, which is not optimal for studying RNA splicing. Others have suggested that the ideal length for splicing junction detection is 100 bp, so that sequences can be uniquely identified on both sides of the junction (Chhangawala et al., 2015). Additionally, King et al. mentioned that the power to map a 10% QTL with 100 DSPR lines is potentially about 10% (King et al., 2012). Therefore, it is challenging to map sQTLs, especially when their effects are relatively small, with only 79 RILs.

Keeping these issues in mind, we used two ways to detect sQTLs: (1) we used the fraction of reads in a transcript that falls in each exon as the quantitative trait (Figure 1B); and (2) we used the differential transcript isoform dosage in the same gene as the quantitative trait (Figure 1C, see section Materials and Methods). Exon/transcript fraction captures changes in reads within each exon, while the second method captures events where both exon reads and transcript reads change, might be missed by the former strategy.

**Figure 4:**
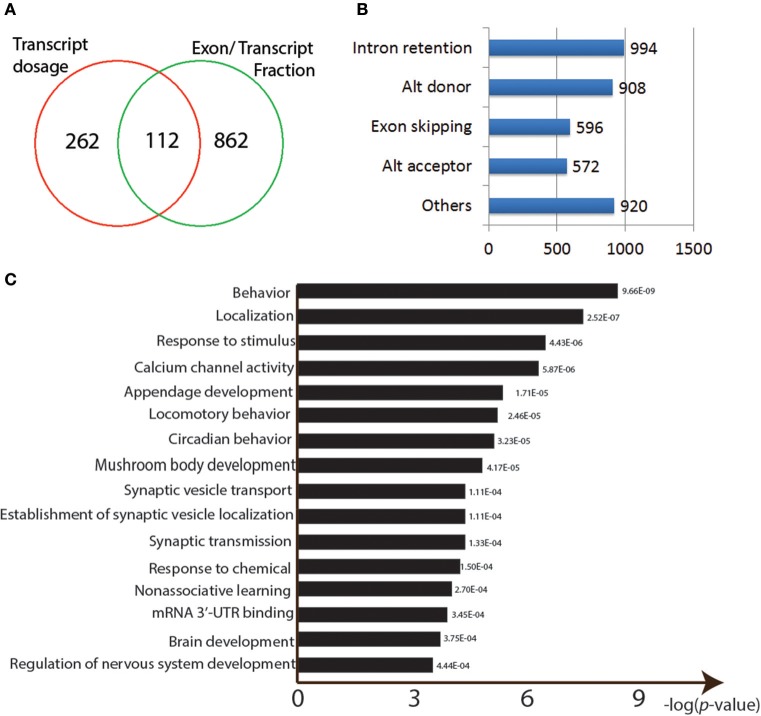
Properties of the Pb-responsive sQTLs. **(A)** Venn graph showing the overlapping sQTLs targeted between two methods. **(B)** Numbers of different AS events found among the identified sQTLs. **(C)** GO enrichment analysis for the sQTLs.

Here, we used ANOVA analysis to detect Pb-responsive sQTLs (see section Materials and Methods; Hoaglin and Welsch, 1978). In total, we obtained 974 potential Pb-responsive sQTLs by calculating exon/transcript fraction and 374 by isoform dosage, with 112 shared ones (*p* < 0.0001, FDR < 0.39; Figure 4A; Supplementary Table S1).

We then used the alternative splicing transcriptional landscape visualization tool (ASTALAVISTA; Foissac and Sammeth, 2007) to determine the types of events represented by the entire set of sQTLs (Figure 4B). The four main AS types were intron retention (*n* = 994), Alternative donor site usage (*n* = 908), exon skipping (*n* = 596), and alternative acceptor site usage (*n* = 572; Figure 4B).

The identified sQTLs were also run through a GO enrichment analysis. The top enriched categories were “behavior” (*p* = 9.66E-09) and “response to stimulus” (*p* = 4.43E-06) and “calcium channel activity” (*p* = 5.87E-06; Figure 4C). Neural developmental related GO categories were also among the most significant: “mushroom body development” (*p* = 4.17E-05), “synaptic vesicle transport” (*p* = 4.17E-05), “non-associative learning” (*p* = 2.70E-04), “brain development” (*p* = 3.75E-04), and “regulation of nervous system development” (*p* = 4.44E-04) (Figure 4C). Other over-represented GO categories include “locomotory behavior” (*p* = 2.46E-05), “response to chemical” (*p* = 1.50E-04), and “mRNA 3′-UTR binding” (*p* = 3.45E-04) (Figure 4C).

**Figure 5:**
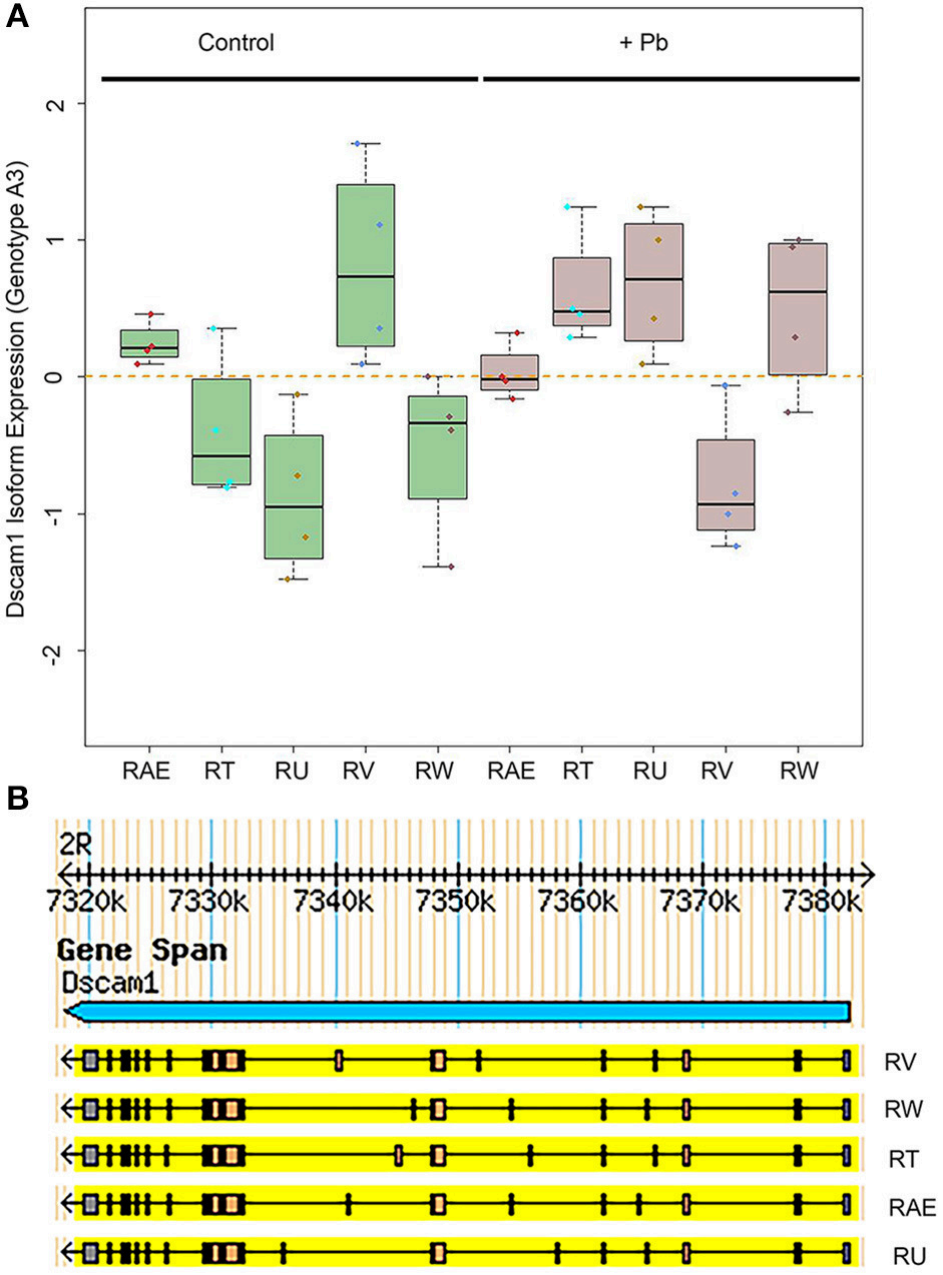
Differential *Dscam1* isoform expression upon lead exposure. **(A)** Green boxes represent control samples and gray boxes represent Pb-treated samples. The RefSeq names of the significantly different alternative transcripts are shown on the X axis. **(B)** UCSD Genome Browser version of 5 isoforms of Dscam1 whose levels are significantly altered by Pb.

**Figure 6:**
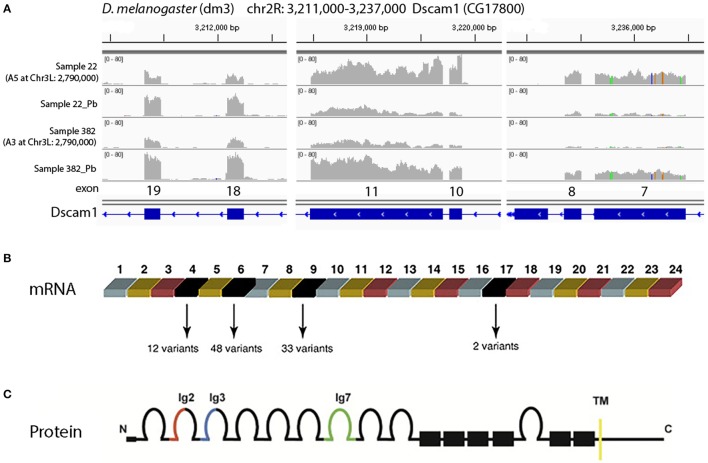
Visualization of different exon usage by RNA-seq w/o lead treatment. **(A)** Sample 22, one of the examples that were originally from A5 strain at Chr3L: 2,790,000, has reduced expression of exon 7, 8, 10, and 11 with lead treatment, while sample 382, one of the examples that were originally from A3 at the same locus, has increased expression of the same exons. However, not all exons share the same feature. For exon 18 and 19, read counts were all increased after lead exposure. **(B)** Dscam1 gene structure showing variant exons 4, 6, 9, and 17. One of each variant exon is used in each transcript, for a theoretical 12 × 48 × 33 × 2 = 38,016 possible mRNAs. **(C)** Dscam1 protein structure showing 10 immunoglobulin (Ig) domains and one transmembrane (TM) domain.

One of the most significant sQTLs is *Dscam1* linked with Chr 3L: 2,790,000 (FDR < 0.1%). Transcript alternative splicing patterns from the A3 parent were altered significantly, while others were not (Figure 5A). In samples that were inherited from A3 parent at Chr 3L: 2,790,000 locus, RT, RU, and RW isoforms were upregulated after lead exposure, RV was downregulated, while RAE was remained steadily. This suggested that A3 strain responded to lead poisoning differently from the rest of the parents by altering usage of the various isoforms. The structures of the alternative spliced isoforms of RT, RU, RW, RV, and RAE are shown (Figure 5B). In order to explore further into the exon usage in *Dscam1*, we used the Integrative Genomics Viewer (IGV) (Thorvaldsdóttir et al., 2013), which is a popular visualization tool for integrated genomic data. We noticed that reads for exon 7, 8, 10, and 11 were increased after lead treatment in A3 samples, while in other samples the expression change was in the reversed direction (Figure 6A). However, not all exons were affected in the same way. For example, read counts for exon 18 and 19 in all samples were upregulated after lead exposure (Figure 6A). We note that it is not the variable exons that are alternatively spliced by the sQTLs we identified in *Dscam1*, but rather the invariant exons that are downstream of the variant exons (Figure 6B). It is not known whether changing the levels of the invariant exons in *Dscam1* would alter the splicing of the variant exons, which encode a possible 38,021 different protein products (Figure 6C). Nevertheless, it's possible that differential exon usage of Dscam1, and the other genes with lead-regulated sQTL, are a part of the compensatory pathway after lead poisoning, but future research is needed to tackle this problem.

### Identification of *trans*-splicing QTLs

**Figure 7:**
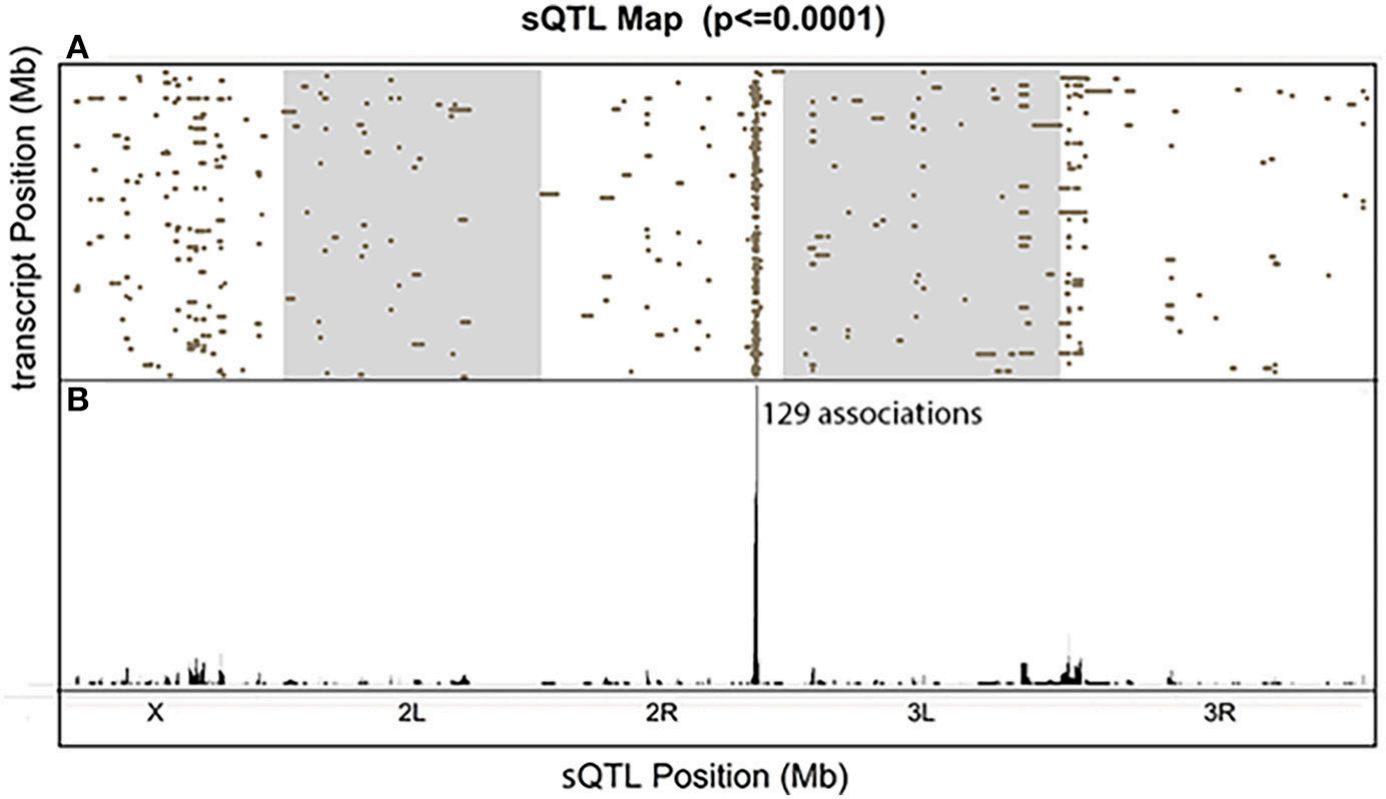
The Comprehensive sQTL Map. **(A)** All significant signals were shown and each dot represents one association between the sQTL location on the x-axis and transcript location on the y-axis. The y-axis represents the ~120 megabase (Mb) euchromatin (non-repetitive) sequence of *Drosophila melanogaster*, from the tip of the X to the dot 4th chromosome. **(B)** The sum of all the associated dots for each genomic location.

We visualized the distribution of all the significant associations with a comprehensive sQTL map (Figure 7). Each dot represents one significant association between the genetic locus shown on the x-axis and the transcript on the y-axis (FDR < 0.39). Among the scattered dots, there was one prominent vertical band, which we call a *trans*-sQTL hotspot, located at Chr3L:18,810,000, consisting of a high density of dots representing 129 different genes located throughout the genome. The *trans*-sQTL hotspot located at Chr3L:18,810,000 is a genomic locus that regulates a cluster of transcripts regardless of their loci. This *trans*-sQTL hotspot is similar to what has been observed in many eQTL studies, where genomic loci that are linked with abnormally high numbers of eQTLs are called *trans*-eQTL hotspots (Joo et al., 2014; King et al., 2014) or *trans*-eQTL bands (Rockman and Kruglyak, 2006). Intriguingly, the *trans*-sQTL hotspot at Chr3L:18,810,000 locus is Pb-responsive (*p* < 1E-10), because it is only present in the presence of Pb and not in the control data. We also found that “ion transport domain” was the topmost GO category (*FDR* = *0.03*) for the list of 129 genes. The six genes in this category and their ion transport functions are listed in Table 1. Interestingly, 58 of the 129 genes (45%) undergo RNA editing, which is almost 10-fold higher than the average (Supplementary Table S2).

**Table 1:** Ion transport genes in trans-splicing QTL.

**Genes**	**Description**	**References**
Shaw	Shaker cognate w (Shaw) is a voltage-gated potassium channel (Kv3.1) that mediates a non-inactivating potassium current open at resting membrane potential. Shaw regulates circadian rhythms and is in a pathway with a Na+ K+ Ca2+ Co-transporter.	Hodge et al., 2005; Hodge and Stanewsky, 2008
Slo	Slowpoke (Slo) the structural alpha subunit of a BK (“maxi K”) calcium-activated potassium channel. Slo channels function to regulate neurotransmitter release at the synapse and maintain electrical excitability in neurons and muscle cells.	Pallanck and Ganetzky, 1994; Lee et al., 2014
Itp-R83A	Inositol 1,4,5,-tris-phosphate receptor (Itp-r83A) is an intracellular ligand gated calcium channel. It functions downstream of G-protein coupled receptors.	Bollepalli et al., 2017
Ca-Alpha1D	Ca2+-channel protein α1 subunit D (Ca-α1D) is the pore-forming α subunit of an L-type voltage-gated Ca2+ channel expressed in neurons.	Hara et al., 2015
CG42260	CG42260 is predicted to form the beta subunit of a cyclic nucleotide-gated cation channel.	Walkinshaw et al., 2015
Wtrw	Water witch (wtrw) is a calcium ion transporter that is involved in sensory perception of sound; and response to humidity.	Liu et al., 2007

## Discussions and conclusions

In this paper, we used two methods to detect Pb-responsive sQTLs. The first one, which is the fraction of exon reads to the transcript reads, searches splicing events on exon levels and all types of genetic–related AS events that cause change either in exon or in transcript after lead treatment will be selected. The other method compares the transcript counts among isoforms. It is on the transcript levels and will select those have differential isoform dosage after lead exposure. The combination of two methods resulted in 1,236 potential Pb-responsive sQTLs.

**Figure 8:**
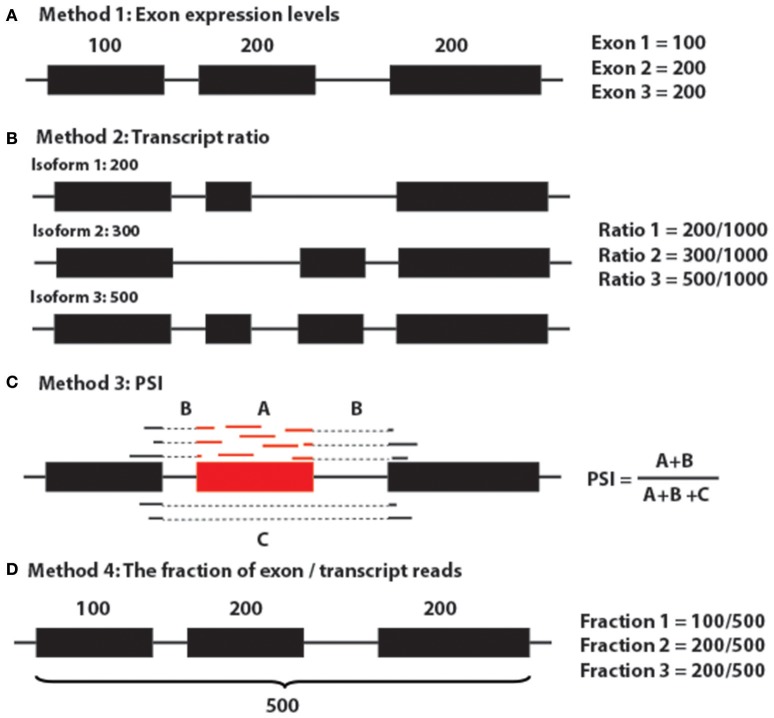
Major methods for detecting sQTLs. **(A)** Method 1: Exon expression levels. **(B)** Method 2: Transcript ratio. **(C)** Method 3: PSI. **(D)** Method 4: The fraction of exon/transcript reads.

Generally, to target sQTLs, there are five major approaches (Figure 8; Gymrek, 2014): (1) use exon expression profiles as the quantitative trait and this could also be referred to as exon QTLs (Montgomery et al., 2010; Lappalainen et al., 2013; Gymrek, 2014); (2) use the proportion of each transcript quantification of the sum of all transcripts per gene (Figure 5A; Lappalainen et al., 2013; Battle et al., 2014; Gymrek, 2014); (3) use percent spliced in (PSI) (Lappalainen et al., 2013; Zhao et al., 2013; Gymrek, 2014; Kurmangaliyev et al., 2015); (4) use the fraction of reads in a gene that falls in a given exon as the phenotype (Figure 6; Pickrell et al., 2010; Gymrek, 2014); and (5) multivariate approaches, such as sQTLseekeR (Gymrek, 2014; Monlong et al., 2014). The sQTLseekeR is a multivariate model called for each gene consisting of the relative abundance of each transcript (Monlong et al., 2014). It calculated the variability of splicing ratios of a gene across samples by using a MANOVA-like distance-based approach and then compared the variability of the splicing ratios within genotypes with the variability among genotypes. We have run our data through the sQTLseekeR pipeline. However, no significant association was returned. One of the potential explanation for this result is that the sQTLseekeR was originally designed to incorporate the genotypes as SNP information (0 for ref/ref, 1 for ref/mutated, 2 for mutated/mutated), but our genotype data, which represent the original parents of evenly distributed genomic locations (A1–A8, representing 8 parents), pose potential challenges to process the data (Monlong et al., 2014).

Most of the sQTL studies were performed in human cell lines. One study by Kurmangaliyev et al. which has claimed to be the first sQTL study in *Drosophila*, searched for genotype-specific alternative splicing donor/acceptor sites by using 81 *Drosophila* hybrid strains generated by crossing natural populations to a single inbred reference line (Kurmangaliyev et al., 2015). They found 59 AS donor/acceptor events by performing 120,240 association tests (Kurmangaliyev et al., 2015). In our study, we ran over 1,255,422,008 association tests (106,681 exon/transcript reads and 11,768 genomic loci) and our detection should not only include alternative donor/acceptor splicing but also other types of AS events.

The identification of *Dscam1* as one of the most significant sQTLs helps to further understand the isoform usage and changes after Pb exposure. We observed the expression alterations in *Dscam1* both at the exon level and at the transcript level after lead poisoning. However, we have few ideas on how to interpret these phenomena: why changes occurred after lead exposure and whether they could contribute to the neural developmental damage. Among the previous studies, Schmucker et al. have shown that the overexpression of one *Dscam1* isoform resulted in strong dominant phenotypes in mushroom body neurons (Schmucker et al., 2000). Some others have found that the diversity of *Dscam1* isoforms allows neurons to have differential patterns on their cell membrane and interaction was performed through isoform-specific hemophilic binding (Wojtowicz et al., 2004; Zipursky and Grueber, 2013; Armitage et al., 2015; Tadros et al., 2016). Schmucker and Flanagan also suggested either that different neurons express different *Dscam1* isoforms or that isoforms need to be present at a precise concentration or a certain development time period (Schmucker and Flanagan, 2004). More analysis is needed and future studies might consider combining sQTL analysis with other molecular and cellular experiments to further explore lead neurotoxicology.

One interesting finding is the preliminary identification of a *trans*-sQTL hotspot that is apparently regulated by Pb. This *trans*-sQTL hotspot, located at Chr3L:18,810,000, includes 129 genes that are significantly enriched in the gene ontology category “ion channel domain” (Table 1). One possible explanation for the *trans*-sQTL hotspot is that this locus encodes a splicing factor whose activity is altered by Pb exposure. Since Pb^2+^ is a cation, the induction of alternative splicing of a cation channels could lead an increase in the elimination of lead from neurons, for instance. While this hypothesis is provocative, it would need to be confirmed experimentally by determining whether the Pb-induced isoforms of these alternatively spliced calcium channels have an increased ability to transport Pb^2+^ out of neurons. Further validation is also needed to confirm that the Dscam1 alternative splicing isoforms that we identified are splicing QTL (Figure 5). However, since used the total reads in each isoform in our quantitative trait mapping analyses, and the alternative exons in each isoform are spread throughout the ~7 kb processed mRNA, the only way to confirm the Dscam1 isoforms is by long RNA-seq analyses. Recently, this was done by the Graveley lab for Dscam1 using the Minion™ nanopore sequencer (Oxford Nanopore, Inc.; Bolisetty et al., 2015), which can sequence tens or even hundreds of kb long cDNA molecules. Attempts by our laboratory to generate long RNA-seq runs of Dscam1 so far have been unsuccessful, but we continue to pursue this approach.

To our knowledge, this study is the first to link splicing QTL analysis with environmental toxin in *Drosophila*. However, there are multiple limitations in our study, which prevent us from reaching more meaningful results: (1) the RNA-seq data were prepared as 50 bp paired-end. However, 100 bp paired-end reads were considered to enhance splicing junction detection significantly (Chhangawala et al., 2015). (2) Both methods in this paper rely on known transcript annotation and transcript level quantifications and these are not widely trusted yet (Gymrek, 2014).

In conclusion, we have shown that sQTL analysis is a useful way in understanding alternative splicing mechanisms and the neuro-toxicology of environmental toxin. We have discovered widespread genetic variation affecting the splicing events. Our characterization of causal regulatory variation sheds light on the mechanisms of neurotoxicity of lead, and allows us to infer putative causal variants for hundreds of potential environmental toxic-associated loci.

## Author contributions

DR is the PI and supervised all aspects of the paper. WQ conducted the research and wrote the paper. KG helped with the bioinformatics and writing. RP helped with the bioinformatics and writing.

### Conflict of interest statement

The authors declare that the research was conducted in the absence of any commercial or financial relationships that could be construed as a potential conflict of interest.
